# Complete genome sequence of *Deinococcus maricopensis* type strain (LB-34^T^)

**DOI:** 10.4056/sigs.1633949

**Published:** 2011-04-25

**Authors:** Rüdiger Pukall, Ahmet Zeytun, Susan Lucas, Alla Lapidus, Nancy Hammon, Shweta Deshpande, Matt Nolan, Jan-Fang Cheng, Sam Pitluck, Konstantinos Liolios, Ioanna Pagani, Natalia Mikhailova, Natalia Ivanova, Konstantinos Mavromatis, Amrita Pati, Roxane Tapia, Cliff Han, Lynne Goodwin, Amy Chen, Krishna Palaniappan, Miriam Land, Loren Hauser, Yun-Juan Chang, Cynthia D. Jeffries, Evelyne-Marie Brambilla, Manfred Rohde, Markus Göker, J. Chris Detter, Tanja Woyke, James Bristow, Jonathan A. Eisen, Victor Markowitz, Philip Hugenholtz, Nikos C. Kyrpides, Hans-Peter Klenk

**Affiliations:** 1 DSMZ – German Collection of Microorganisms and Cell Cultures GmbH, Braunschweig, Germany; 2 DOE Joint Genome Institute, Walnut Creek, California, USA; 3 Los Alamos National Laboratory, Bioscience Division, Los Alamos, New Mexico USA; 4 Biological Data Management and Technology Center, Lawrence Berkeley National Laboratory, Berkeley, California, USA; 5 Lawrence Livermore National Laboratory, Livermore, California, USA; 6 HZI – Helmholtz Centre for Infection Research, Braunschweig, Germany; 7 University of California Davis Genome Center, Davis, California, USA; 8 Australian Centre for Ecogenomics, School of Chemistry and Molecular Biosciences, The University of Queensland, Brisbane, Australia

**Keywords:** aerobic, non-motile, Gram-positive, radiation-resistant, mesophilic, chemoorganotrophic, *Deinococcaceae*, GEBA

## Abstract

*Deinococcus maricopensis* (Rainey and da Costa 2005) is a member of the genus *Deinococcus,* which is comprised of 44 validly named species and is located within the deeply branching bacterial phylum *Deinococcus*–*Thermus*. Strain LB-34^T^ was isolated from a soil sample from the Sonoran Desert in Arizona. Various species of the genus *Deinococcus* are characterized by extreme radiation resistance, with *D. maricopensis* being resistant in excess of 10 kGy. Even though the genomes of three *Deinococcus* species, *D. radiodurans*, *D. geothermalis* and *D. deserti,* have already been published, no special physiological characteristic is currently known that is unique to this group. It is therefore of special interest to analyze the genomes of additional species of the genus *Deinococcus* to better understand how these species adapted to gamma- or UV ionizing-radiation. The 3,498,530 bp long genome of *D. maricopensis* with its 3,301 protein-coding and 66 RNA genes consists of one circular chromosome and is a part of the *** G****enomic* *** E****ncyclopedia of* *** B****acteria and* *** A****rchaea * project.

## Introduction

Strain LB-34^T^ (= DSM 21211 = NRRL B-23946 = LMG 22137) is the type strain of *Deinococcus maricopensis* [1]. In addition to the type strain LB-34^T^, two more strains of this species, KR 1 and KR 23, were characterized by Rainey *et al.* [1]. The generic name derives from the Greek words ‘deinos’ meaning ‘strange or unusual’ and ‘coccus’ meaning ‘a grain or berry’ [2]. The species epithet is derived from the Neo-Latin word ‘maricopensis’ referring to the Maricopa Nation, a native tribe in Arizona [1]. Strain LB 34^T^ was isolated from desert soil in Arizona and described by Rainey *et al.* in 2005 [1]. The genus *Deinococcus* was proposed in 1981 by Brooks and Murray [2] to separate the distinct radiation-resistant species from the genus *Micrococcus* in which those species were originally classified. With the description of *Deinobacter grandis* by Oyaizu *et al*. [3], a second genus was placed to the family *Deinococcaceae*, and in 1997 Rainey *et al*. proposed to transfer *Deinobacter* to the genus *Deinococcus*, based on investigations of the phylogenetic diversity of the *Deinococci* as determined by 16S rRNA gene sequence analysis. In conclusion, an emended description of the genus *Deinococcus* was published, showing that the cells can be spherical or rod-shaped [4]. Members of the genus *Deinococcus* were isolated from various environmental habitats including air [5-7], arid soil [1,8-12], water and activated sludge [13-15], alpine environments [16], rhizosphere [17], Antarctica [18], hot springs [19], aquifer [20], marine fish [21] and radioactive sites [22]. Here we present a summary classification and a set of features for *D. maricopensis* LB-34^T^, together with the description of the complete genomic sequencing and annotation.

## Classification and features

A representative genomic 16S rRNA sequence of strain LB-34^T^ was compared using NCBI BLAST under default settings (e.g., considering only the high-scoring segment pairs (HSPs) from the best 250 hits) with the most recent release of the Greengenes database [23] and the relative frequencies, weighted by BLAST scores, of taxa and keywords (reduced to their stem [24]) were determined. The single most frequent genus was *Deinococcus* (100.0%) (114 hits in total). Regarding the three hits to sequences from members of the species, the average identity within HSPs was 99.9%, whereas the average coverage by HSPs was 97.6%. Regarding the 77 hits to sequences from other members of the genus, the average identity within HSPs was 91.5%, whereas the average coverage by HSPs was 60.5%. Among all other species, the one yielding the highest score was *D. radiodurans*, which corresponded to an identity of 91.2% and an HSP coverage of 88.0%. The highest-scoring environmental sequence was AY905380 ('Extensive ionizing-radiation-resistant recovered sonoran and description nine new species genus *Deinococcus* obtained single mixed agricultural/open desert soil clone L14-471'), which showed an identity of 98.1% and a HSP coverage of 70.2%. The five most frequent keywords within the labels of environmental samples which yielded hits were 'skin' (7.7%), 'litholog/stream' (2.8%), 'fossa' (2.4%), 'microbi' (2.4%) and 'forearm' (2.1%) (136 hits in total). Environmental samples which yielded hits of a higher score than the highest scoring species were not found.

**Figure 1:**
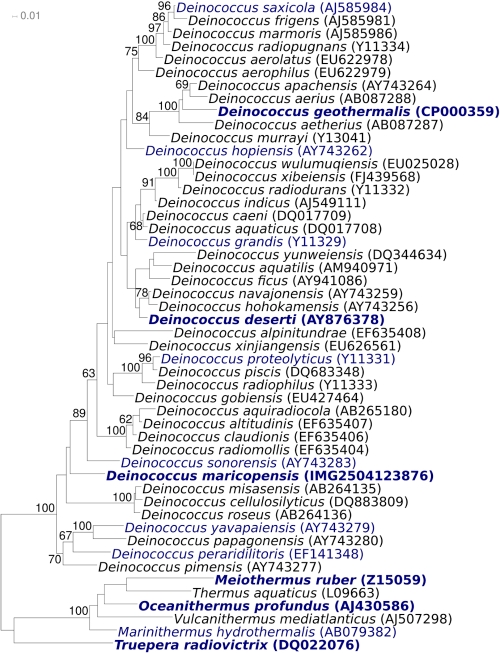
Phylogenetic tree highlighting the position of *D. maricopensis* relative to the other type strains within the family *Deinococcaceae*. The tree was inferred from 1,382 aligned characters [25,26] of the 16S rRNA gene sequence under the maximum likelihood criterion [27] and rooted in accordance with the current taxonomy. The branches are scaled in terms of the expected number of substitutions per site. Numbers above branches are support values from 1,000 bootstrap replicates [28] if larger than 60%. Lineages with type strain genome sequencing projects registered in GOLD [29] are shown in blue, and published genomes in bold [30-34]. The genome of *D. radiodurans* published by White *at al.* in 1999 [35] later turned out not to be from the type strain [36].

Figure 1 shows the phylogenetic neighborhood of *D. maricopensis* LB-34^T^ in a 16S rRNA based tree. The sequences of the four identical 16S rRNA gene copies in the genome differ by one nucleotide from the previously published 16S rRNA sequence (AY743274).

**Figure 2:**
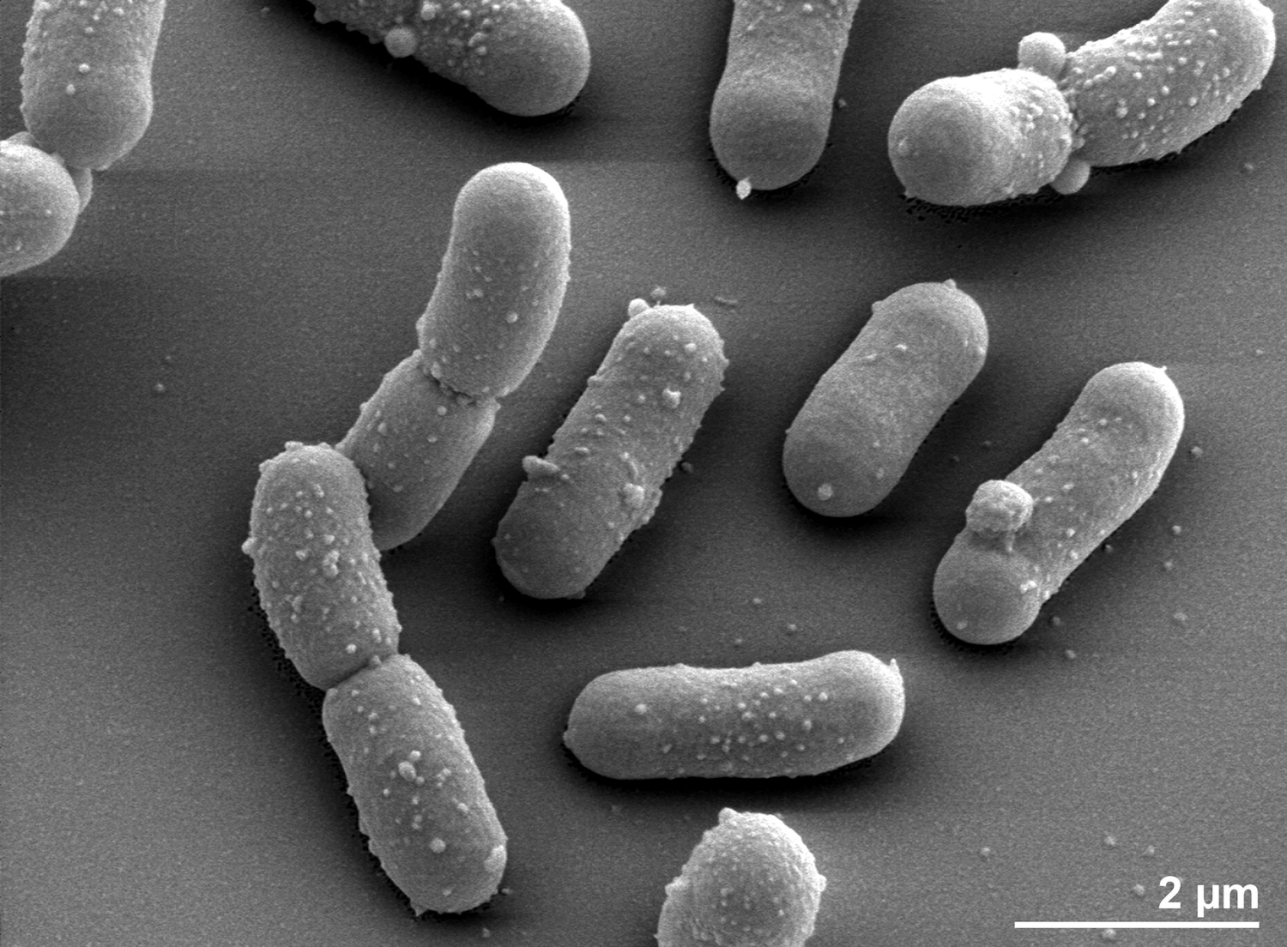
Scanning electron micrograph of *D. maricopensis* LB-34^T^

The cells of *D. maricopensis* are rod-shaped, up to 6 µm in length and 2.0 µm wide (Figure 2). *D. maricopensis* is a Gram-positive, non-spore-forming bacterium (Table 1). Colonies on Rich medium are orange to pink. The cells are non-motile. The organism is chemoorganotrophic [1]. The temperature range for growth is 10° to 45°C, with an optimum at 40°C [1]. Cytochrome oxidase and catalase activity have been observed [1]. Strains may utilize L-arabinose, cellobiose, galactose, glucose, mannose, maltose, sucrose, trehalose, glucosamine, glycerol, malate, asparagine, aspartate, glutamate, L-glutamine, ornithine and proline. Fructose can be used by strain KR23, but not by strain LB-34^T^ [1]. Strain LB-34^T^ showed similar levels of desiccation tolerance of up to four weeks as compared to *D. radiodurans* strain R1^T^. Strain LB-34^T^ is resistant to > 10kGy, but more sensitive to ionizing radiation than strain *D. radiodurans* R1^T^ [1].

**Table 1:** Classification and general features of *D. maricopensis* LB-34^T^according to the MIGS recommendations [37].

MIGS ID	Property	Term	Evidence code
	Current classification	Domain *Bacteria*	TAS [38]
		Phylum *Deinococcus*-*Thermus*	TAS [39]
		Class *Deinococci*	TAS [40,41]
		Order *Deinococcales*	TAS [4]
		Family *Deinococcaceae*	TAS [2,4]
		Genus *Deinococcus*	TAS [2,4]
		Species *Deinococcus maricopensis*	TAS [1,42]
		Type strain LB-34	TAS [1]
	Gram stain	positive	TAS [1]
	Cell shape	rods	TAS [1]
	Motility	non-motile	TAS [1]
	Sporulation	none	TAS [1]
	Temperature range	mesophile, 10°C–45°C	TAS [1]
	Optimum temperature	40°C	TAS [1]
	Salinity	not reported	
MIGS-22	Oxygen requirement	aerobic	TAS [1]
	Carbon source	carbohydrates	TAS [1]
	Energy metabolism	chemoorganotroph	TAS [1,2]
MIGS-6	Habitat	soil	TAS [1]
MIGS-15	Biotic relationship	free-living	NAS
MIGS-14	Pathogenicity	none	NAS
	Biosafety level	1	TAS [43]
	Isolation	soil	TAS [1]
MIGS-4	Geographic location	Sonoran Desert, Arizona, USA	TAS [1]
MIGS-5	Sample collection time	1999	NAS
MIGS-4.1	Latitude	32.93	NAS
MIGS-4.2	Longitude	-112.30	NAS
MIGS-4.3	Depth	not reported	
MIGS-4.4	Altitude	not reported	

Evidence codes - IDA: Inferred from Direct Assay (first time in publication); TAS: Traceable Author Statement (i.e., a direct report exists in the literature); NAS: Non-traceable Author Statement (i.e., not directly observed for the living, isolated sample, but based on a generally accepted property for the species, or anecdotal evidence). These evidence codes are from of the Gene Ontology project [44]. If the evidence code is IDA, then the property was directly observed by one of the authors or an expert mentioned in the acknowledgements.

### Chemotaxonomy

The major cellular fatty acids of the strain LB-34^T^ were identified as *iso*-C_15:0_, *iso*-C_17:0_ and C_16:0_. Menaquinone 8 (MK-8) was determined as the major respiratory quinone of the strain. Phosphoglycolipid and glycolipid pattern are similar to those of other *Deinococcus* species [1]. No data are available for strain LB-34^T^ showing the peptidoglycan type of the cell wall.

## Genome sequencing and annotation

### Genome project history

This organism was selected for sequencing on the basis of its phylogenetic position [45], and is part of the *** G****enomic* *** E****ncyclopedia of* *** B****acteria and* *** A****rchaea * project [46]. The genome project is deposited in the Genomes On Line Database [29] and the complete genome sequence is deposited in GenBank. Sequencing, finishing and annotation were performed by the DOE Joint Genome Institute (JGI). A summary of the project information is shown in Table 2.

**Table 2:** Genome sequencing project information

**MIGS ID**	**Property**	**Term**
MIGS-31	Finishing quality	Finished
MIGS-28	Libraries used	Three genomic libraries: one 454 pyrosequence standard library, one 454 PE library (7 kb insert size), one Illumina library
MIGS-29	Sequencing platforms	Illumina GAii, 454 GS FLX Titanium
MIGS-31.2	Sequencing coverage	170.9 × Illumina; 75.4 × pyrosequence
MIGS-30	Assemblers	Newbler version 2.3-PreRelease-10-21-2009-gcc-4.1.2-threads, Velvet version 0.7.63, phrap
MIGS-32	Gene calling method	Prodigal 1.4, GenePRIMP
	INSDC ID	CP002454
	Genbank Date of Release	January 20, 2011
	GOLD ID	Gc01597
	NCBI project ID	43461
	Database: IMG-GEBA	2503982045
MIGS-13	Source material identifier	DSM 21211
	Project relevance	Tree of Life, GEBA

### Growth conditions and DNA isolation

*D. maricopensis* LB-34^T^, DSM 21211, was grown in DSMZ medium 736 (Rich Medium) [47] at 28°C. DNA was isolated from 0.5-1 g of cell paste using MasterPure Gram-positive DNA purification kit (Epicentre MGP04100) following the standard protocol as recommended by the manufacturer, with a modification in cell lysis by adding 20 μl lysozyme (100 mg/μl), and 10 μl mutanolysine, achromopeptidase and lysostphine, each, for 40 min at 37°C, followed by one hour incubation on ice after the MPC step. DNA is available through the DNA Bank Network [48,49].

### Genome sequencing and assembly

The genome was sequenced using a combination of Illumina and 454 sequencing platforms. All general aspects of library construction and sequencing can be found at the JGI website [50]. Pyrosequencing reads were assembled using the Newbler assembler version 2.3 (Roche). The initial Newbler assembly consisting of 58 contigs in two scaffolds was converted into a phrap assembly by [51] making fake reads from the consensus, to collect the read pairs in the 454 paired end library. Illumina GAii sequencing data (957.8 Mb) were assembled with Velvet version 0.7.63 [52] and the consensus sequences were shredded into 1.5 kb overlapped fake reads and assembled together with the 454 data. The 454 draft assembly was based on 234.5 Mb 454 draft data and all of the 454 paired end data. Newbler parameters are -consed -a 50 -l 350 -g -m -ml 20. The Phred/Phrap/Consed software package [51] was used for sequence assembly and quality assessment in the subsequent finishing process. After the shotgun stage, reads were assembled with parallel phrap (High Performance Software, LLC). Possible mis-assemblies were corrected with gapResolution [50], Dupfinisher [53], or sequencing cloned bridging PCR fragments with subcloning or transposon bombing (Epicentre Biotechnologies, Madison, WI). Gaps between contigs were closed by editing in Consed, by PCR and by Bubble PCR primer walks (J.-F.Chang, unpublished). A total of 255 additional reactions were necessary to close gaps and to raise the quality of the finished sequence. Illumina reads were also used to correct potential base errors and increase consensus quality using a software Polisher developed at JGI [54]. The error rate of the completed genome sequence is less than 1 in 100,000. Together, the combination of the Illumina and 454 sequencing platforms provided 246.3 × coverage of the genome. The final assembly contained 872,337 pyrosequence and 16,604,657 Illumina reads.

### Genome annotation

Genes were identified using Prodigal [55] as part of the Oak Ridge National Laboratory genome annotation pipeline, followed by a round of manual curation using the JGI GenePRIMP pipeline [56]. The predicted CDSs were translated and used to search the National Center for Biotechnology Information (NCBI) nonredundant database, UniProt, TIGR-Fam, Pfam, PRIAM, KEGG, COG, and InterPro databases. Additional gene prediction analysis and functional annotation was performed within the Integrated Microbial Genomes - Expert Review (IMG-ER) platform [57].

## Genome properties

**Figure 3:**
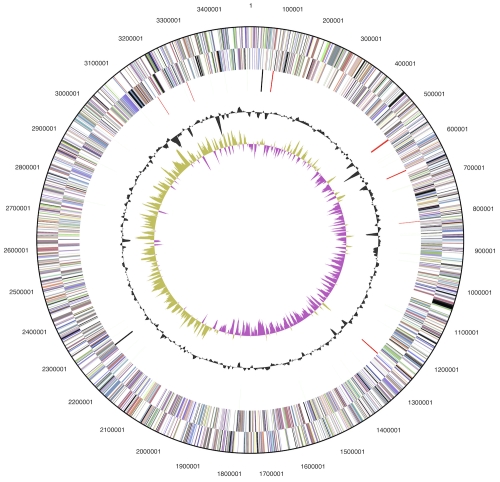
Graphical circular map of the chromosome. From outside to the center: Genes on forward strand (color by COG categories), Genes on reverse strand (color by COG categories), RNA genes (tRNAs green, rRNAs red, other RNAs black), GC content, GC skew.

The genome consists of a 3,498,530 bp long chromosome with a G+C content of 69.8% (Table 3 and Figure 3). Of the 3,367 genes predicted, 3,301 were protein-coding genes, and 66 RNAs; 37 pseudogenes were also identified. The majority of the protein-coding genes (70.3%) were assigned with a putative function while the remaining ones were annotated as hypothetical proteins. The distribution of genes into COGs functional categories is presented in Table 4.

**Table 3:** Genome Statistics

**Attribute**	**Value**	**% of Total**
Genome size (bp)	3,498,530	100.00%
DNA coding region (bp)	3,127,041	89.38%
DNA G+C content (bp)	2,442,849	69.83%
Number of replicons	1	
Extrachromosomal elements	0	
Total genes	3,367	100.00%
RNA genes	66	1.96%
rRNA operons	4	
Protein-coding genes	3,301	98.04%
Pseudo genes	37	1.10%
Genes with function prediction	2,366	70.27%
Genes in paralog clusters	368	10.93%
Genes assigned to COGs	2,412	71.64%
Genes assigned Pfam domains	2,495	74.10%
Genes with signal peptides	1,005	29.85%
Genes with transmembrane helices	662	19.66%
CRISPR repeats	0	

**Table 4:** Number of genes associated with the general COG functional categories

**Code**	**value**	**%age**	**Description**
J	160	6.0	Translation, ribosomal structure and biogenesis
A	0	0.0	RNA processing and modification
K	188	7.1	Transcription
L	109	4.1	Replication, recombination and repair
B	2	0.1	Chromatin structure and dynamics
D	29	1.1	Cell cycle control, cell division, chromosome partitioning
Y	0	0.0	Nuclear structure
V	45	1.7	Defense mechanisms
T	195	7.3	Signal transduction mechanisms
M	137	5.2	Cell wall/membrane/envelope biogenesis
N	15	0.6	Cell motility
Z	1	0.0	Cytoskeleton
W	0	0.0	Extracellular structures
U	43	1.6	Intracellular trafficking, secretion, and vesicular transport
O	113	4.3	Posttranslational modification, protein turnover, chaperones
C	125	4.7	Energy production and conversion
G	205	7.7	Carbohydrate transport and metabolism
E	237	8.9	Amino acid transport and metabolism
F	77	2.9	Nucleotide transport and metabolism
H	119	4.5	Coenzyme transport and metabolism
I	105	4.0	Lipid transport and metabolism
P	121	4.6	Inorganic ion transport and metabolism
Q	60	2.3	Secondary metabolites biosynthesis, transport and catabolism
R	334	12.6	General function prediction only
S	238	9.0	Function unknown
-	955	28.4	Not in COGs
